# In Vitro Evaluation of NLS-DTX Activity in Triple-Negative Breast Cancer

**DOI:** 10.3390/molecules27154920

**Published:** 2022-08-02

**Authors:** Karen L. R. Paiva, Marina A. Radicchi, Sônia N. Báo

**Affiliations:** 1 Department of Cell Biology, Institute of Biological Sciences, University of Brasília, Brasília 70910-900, DF, Brazil; karendepaiva@gmail.com (K.L.R.P.); maradicchi.bep@gmail.com (M.A.R.); 2 Postgraduate Program of Molecular Pathology, School of Medicine, University of Brasília, Brasília 70910-900, DF, Brazil; 3 Postgraduate Program of Molecular Biology, Institute of Biological Sciences, University of Brasília, Brasília 70910-900, DF, Brazil

**Keywords:** solid lipid nanoparticle, triple-negative breast cancer, docetaxel

## Abstract

Cancer is one of the most lethal diseases in the world, and the development and improvement of treatments used in cancer therapies are extremely important for a better quality of life for patients. In view of the current problems in drug administration such as low solubility and adverse effects, the activity of a solid lipid nanoparticle containing docetaxel (SLN-DTX), a drug already used in conventional therapies, was evaluated in a cell line (MDA-MB-231) of one of the most aggressive types of breast cancer with the worst prognosis, triple-negative breast cancer. Viability tests indicated that SLN-DTX has a greater dependence on the treatment dose when compared to the free drug, which indicates a more controlled release of the drug, and both reduced viability by around 50% at a concentration of 1 µg/mL after 72 h. Transmission electron microscopy (TEM) and confocal and light microscopy analyses indicated that after treatment the cells enter a mitotic catastrophe, characteristic of antimitotic drugs that usually make cells progress to death or senescence. Cells treated with both DTX and SLN-DTX showed significant inhibition of mobility, 73.6% and 66.5% when treated with SLN-DTX and DTX, respectively, compared to the 11.4% of the control after 72 h, characteristics that are very relevant in tumor development and progression. SLN-DTX demonstrated its great potential as a nanocarrier by maintaining and improving the drug’s action in the MDA-MB-231 cell line.

## 1. Introduction

Cancer is a generic term for a group of diseases that arise from a genetic mutation, an alteration in the cell’s DNA, which receives wrong instructions for its activities, especially those related to growth and division. These altered cells tend to be very aggressive and their uncontrollable growth determines the formation of tumors. When these tumors acquire the ability to invade adjacent parts of the body and spread to other structures of the organism, this process is known as metastasis, which is the main cause of fatal outcomes in cancer cases. Cancer is now one of the main causes of death, estimated at 7.6 million deaths every year globally [1,2,3]. Triple-negative breast cancer (TNBC) constitutes approximately 15–20% of all breast cancer patients [4]. It is more frequent in younger patients, usually premenopausal women under 40 years old. There is also a higher frequency among specific ethnicities such as Latin, African, and African American women. Compared with other subtypes of breast cancer, TNBC is more aggressive, due to the shorter survival time, the high mortality rate, and the likelihood of an early relapse [4,5,6]. TNBC has as one of its main characteristics the lack of expression of estrogen and progesterone receptors and absence of ERBB2 (HER2) overexpression on its membrane. Despite being allocated to a single group, TNBC is very diverse, which is reported to be one of the reasons for the different responses to both traditional and new therapies, with a significant discrepancy between the survival times [4,7].

No molecular targets have been identified in TNBC so far, and chemotherapy remains the therapeutic backbone of the disease. Commercially available cancer drugs exhibit a variety of systemic toxic effects, due to many factors including high toxicity, low bioavailability, high drug dispersion in the body, and absence of drug delivery and controlled release devices [8]. The standard chemotherapy against TNBC is based on an anthracycline-taxane combination, but a significant proportion (30–40%) of patients with early-stage TNBC develop metastatic disease and succumb to cancer, so some modifications in the traditional TNBC chemotherapy regimens are necessary [9,10].

Docetaxel (DTX) is considered one of the important anticancer drugs as it has significant activity against different types of human cancers. However, DTX accumulates in cancer cells at low concentrations due to its lack of selectivity. Consequently, it presents low drug efficiency and a high degree of toxicity [11]. DTX is a semi-synthetic compound of the taxane family, and the main antitumor activity of DTX is to contain the depolymerization and disassembly of the connections of the microtubules [12]. DTX alters the reversibility of the process by binding to *β* tubulin, thereby stabilizing the protein and preventing its depolymerization, and thus altering its cell rearrangement, which at first induces cell cycle arrest of the tumor during mitosis. The low solubility and hydrophobicity of DTX create difficulties in the administration, so Tween 80 and ethanol are usually used with DTX for its solubilization in clinical application. These solubilizers have serious side effects, causing most patients to have severe allergic reactions, such as cumulative fluid retention, hypersensitivity reactions, and nausea [13,14,15].

Consequently, the incorporation of docetaxel into nanoparticles, such as solid lipid nanoparticles (SLNs), is a promising strategy for delivery to cancer cells. SLNs were first introduced as drug carriers in 1991 as an alternative to conventional carriers such as nanoemulsion and liposomes [16]. SLNs are considered a new colloidal drug delivery system that successfully combines the qualities of the liposome and the polymeric nanoparticles. SLNs have the ability to provide solid core stability and also biocompatibility of lipid nanocarriers, avoiding limitations related to liposomes and polymeric nanoparticles, such as long-term stability, toxicity, sterilization, and scale-up [17]. These nanoparticles also offer unique properties, including small size, large surface area, high drug load, enhanced therapeutic efficacy of the loaded drug, biocompatibility, chemical and mechanical stability, and ease of functionalization. Furthermore, they can transport hydrophobic and hydrophilic molecules, have very low toxicity or no toxicity, and increase drug action time through prolonged half-life. In particular, the physiological lipid core within SLNs can protect encapsulated drugs from chemical degradation and increase their physical stability. In addition, it has been reported that SLNs modulate release kinetics, improve blood circulation time, and increase the general therapeutic effectiveness of anticancer drugs [18,19,20].

## 2. Results and Discussion

### 2.1. Cell Viability

**Figure 1:**
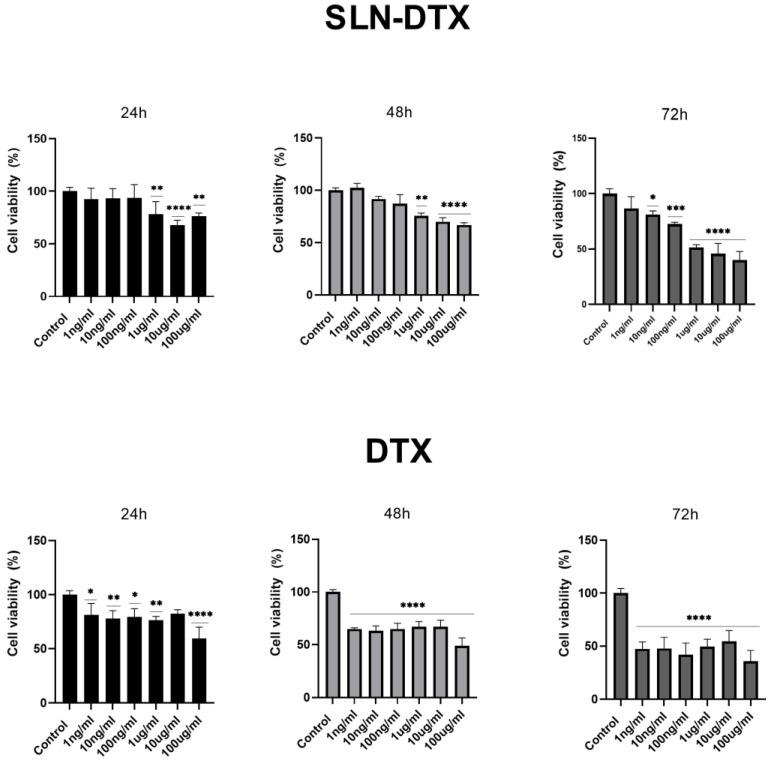
Cell viability analysis of MDA-MB-231 by MTT treated with DTX and SLN-DTX, after 24 h, 48 h, and 72 h. The data represent the mean ± SEM of three independent experiments in triplicate. * *p* < 0.05, ** *p* < 0.01, *** *p* < 0.001 **** *p* < 0.0001 compared to the untreated control.

After 72 h of treatment with SLN-DTX and DTX, the viability of MDA-MB-231 cells was reduced to 50%. In both treatments, the concentration was 1 µg/mL, and this was used in other tests to compare the effects of treatments. However, cells treated with SLN-DTX showed not only a time-dependent reduction in viability, thus ensuring their sustained release, but also a greater dependence on the administered dose, as can be observed in Figure 1. This was not observed for DTX alone.

The characteristics of this nanoparticle were previously described by Da Rocha et al. (2020) [21] in a murine cell line (4T1) and also observed in a human cell line (MDA-MB-231). This confirms the translational activity of this nanoparticle between human and mouse cell lines. Despite the metabolic differences, the two present a similar response, and this amplifies the potential for studies on other types of cancer. This behavior was also observed in other solid lipid nanoparticles such as linalool-loaded solid lipid nanoparticles [8], docetaxel palmitate solid lipid nanoparticles [22], and docetaxel-incorporated lipid nanoparticles [23].

Increased uptake of nanoparticles leads to increased accumulation of intracellular DTX, inducing higher cytotoxic effects. Longer exposure to DTX in the cytosol, due to sustained release, may lead to increased cytotoxicity over time, because DTX is a cell cycle-specific anticancer agent [23].

### 2.2. Cell Morphology

**Figure 2:**
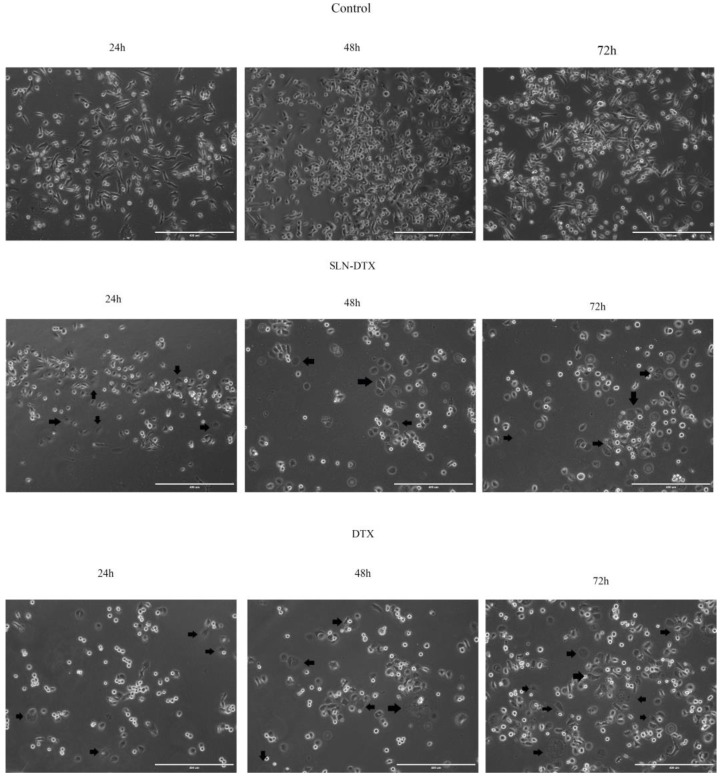
Morphological evaluation of MDA-MB-231 cells at 24 h, 48 h, and 72 h after control and treatment with SLN-DTX and DTX by light microscopy. Arrows indicate treated cells that show changes in the cytoplasm. Scale bar 400 μm.

The evaluation by light microscopy showed, as can be seen in Figure 2, that the control cells maintained their morphology unchanged over time. Cells showed a fusiform shape, there was an increase in cell density when compared to the initial time and the cells remained adhered. After treatment with SLN-DTX, cells lost their standard morphology and became more rounded, with many of them losing their adhesion and decreasing in size. The density of cells was also considerably lower than the control.

**Figure 3:**
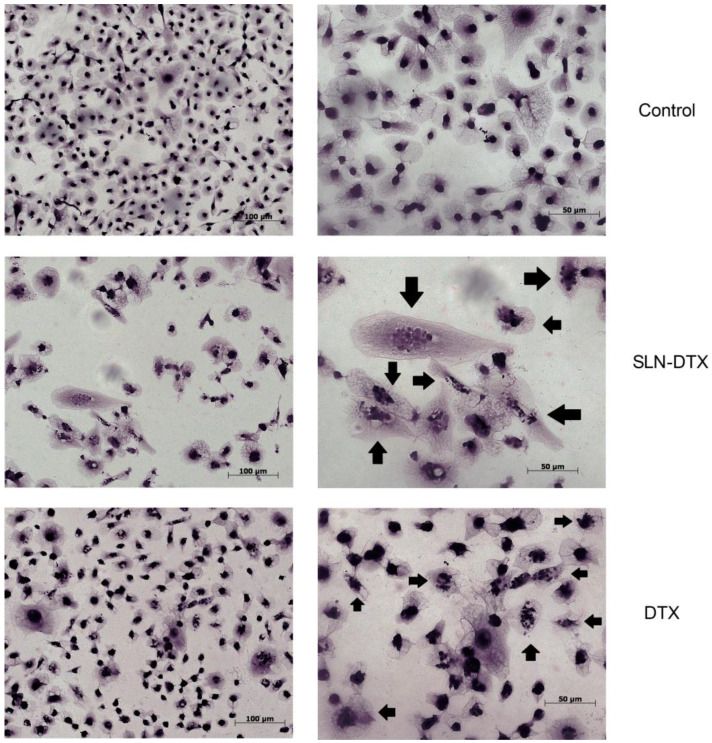
Morphological evaluation of the MDA-MB-231 cell line stained with hematoxylin and eosin after 72 h of treatment with SLN-DTX and DTX observed by light microscopy. Arrows indicate cells that show the altered nucleus, with multinucleation in both treatments.

To better observe the cytoplasmic and nuclear changes caused by the treatment, the cells were stained with hematoxylin and eosin. In Figure 3, it can be observed that there were relevant alterations in the cytoplasm, in which either the cells were drastically reduced or expanded, as also observed in the 4T1 cell line [21]. It is also possible to notice that the nucleus of treated cells became segmented after both treatments, which is characteristic of mitotic catastrophe [24]. 

**Figure 4:**
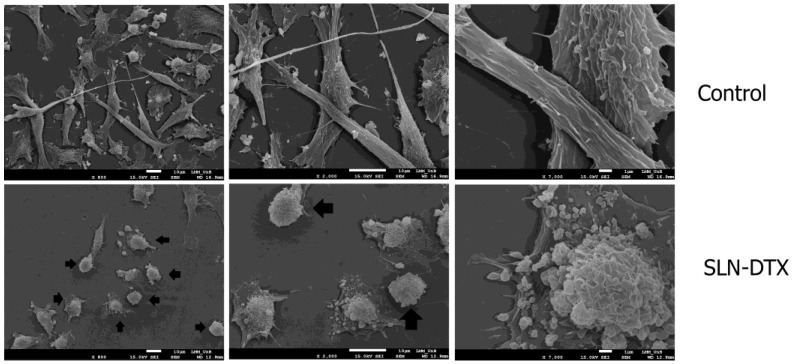
Scanning electron microscopy of MDA-MB-231 cells after 72 h of treatment with SLN-DTX. Arrows indicate cells that have lost their extensions. Changes in cell surface after treatment are visible, as is the reduction in cell density.

The changes observed by light microscopy are emphasized when analyzed by scanning electron microscopy. In Figure 4, the structure of the cell surface was greatly altered after treatment with SLN-DTX. In addition to the reduction of cell density, it is possible to observe changes in the membrane structure. The membrane of the treated cell became rougher, with villi, and there was a reduction of the very characteristic extensions of the cell.

The stability of the beta-tubulin polymerization and depolymerization cycle is important for several cellular functions, among them the maintenance of the cellular structure [25]. It can be seen in light microscopy and SEM that the control cells showed a fusiform shape, while the cells treated with SLN-DTX were more rounded, and they lost their extensions, shrank, and detached from the plate.

### 2.3. Immunostaining of Beta-Tubulin

**Figure 5:**
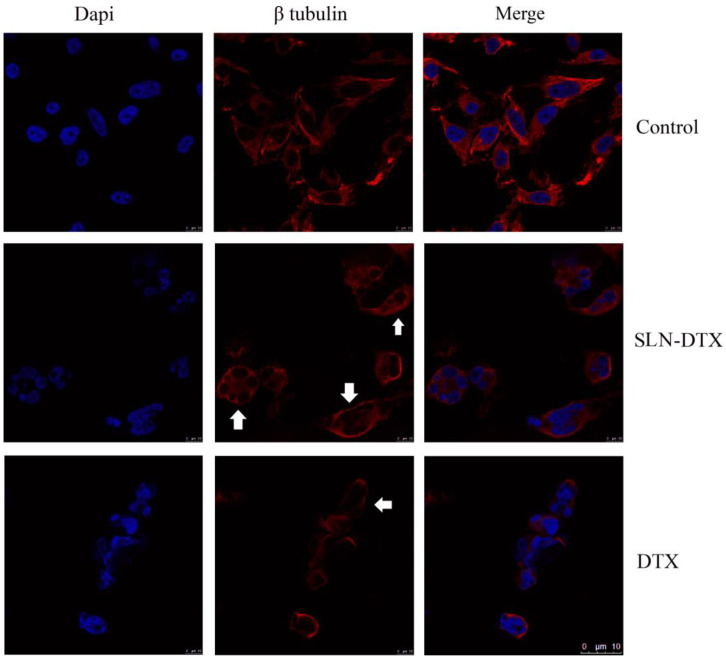
Beta-tubulin immunostaining on MDA-MB-231 after 72 h of treatment. DAPI stained the nucleus in blue, in red is the immunostaining of beta-tubulin and the overlap of the two. The arrows indicate the structure of beta-tubulin, delimiting the segmentations of the nucleus. Scale bar 10 μm.

Microtubules are the major filamentous components of the eukaryotic cytoskeleton and are essential for all cells, as they control the shape, division, motility, and differentiation of cells [25]. In the absence of microtubule dynamics, the mitotic spindle cannot be formed properly, resulting in prolonged mitotic arrest and subsequent growth arrest and cell death [26]. These compounds bind to microtubules at specific sites, and taxanes interact with *β*-tubulin and stabilize the binding between microtubules [27]. With immunostaining, it can be seen in Figure 5 that beta-tubulin was found in the cytoplasm delimiting the nuclear structure. The cell nucleus in treated groups was segmented, as noted in previous analyses. The structural arrangement of the cell changed with the reduction of cellular extensions after treatments.

Failures in the control of microtubules are often associated with chromosome breakage and impaired mitokinesis, which leads to gross nuclear alterations (micronucleation and multinucleation) that constitute the most prominent morphological features of the mitotic catastrophe [28].

### 2.4. Ultra-Structural Analyses

**Figure 6:**
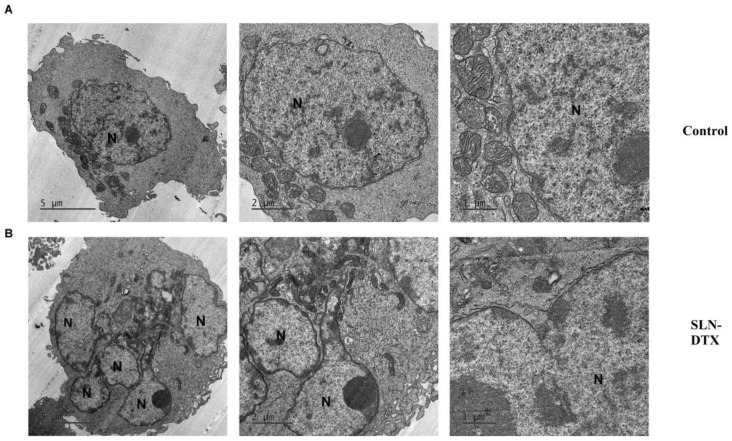
Transmission electron microscopy of MDA-MB-231 after 72h treatment with SLN-DTX and the control. (**A**) MDA-MB-231 control cells and (**B**) MDA-MB-231 treated with SLN-DTX after 72h. N: nucleus. Observation of segmented nucleus, characteristic multinucleation after treatment with SLN-DTX. At higher magnification, it is possible to observe that the nuclear segments are delimited by the nuclear envelope.

In the ultra-structural analyses of the cells treated with SLN-DTX for 72 h in Figure 6, the nucleus became segmented, and at higher magnification the delimitation of these segments by the nuclear envelope became clear. The cells presented fewer cell membrane extensions, and this structure was altered as the treatment targeted tubulin, which composes the cell’s cytoskeleton.

TEM images corroborate other microscopy analyses, showing that mitotic catastrophe occurred after treatment with SLN-DTX, which was behavior not observed in control cells. Studies indicate that mitotic catastrophe constitutes a cellular mechanism used to preserve genomic stability, and it can be leveraged to selectively kill cancerous cells [28]. This suggests the possibility that the checkpoint necessary for chromosomal segregation in mitotic processes can affect tumor cells more severely than normal cells [29]. Other nanoformulations have already induced the same behavior after treatment with taxanes, corroborating those disturbances in microtubule dynamics by polarization stabilization leading to mitotic catastrophe [5].

### 2.5. Internalization of SNL by TEM and Confocal Microscopy

**Figure 7:**
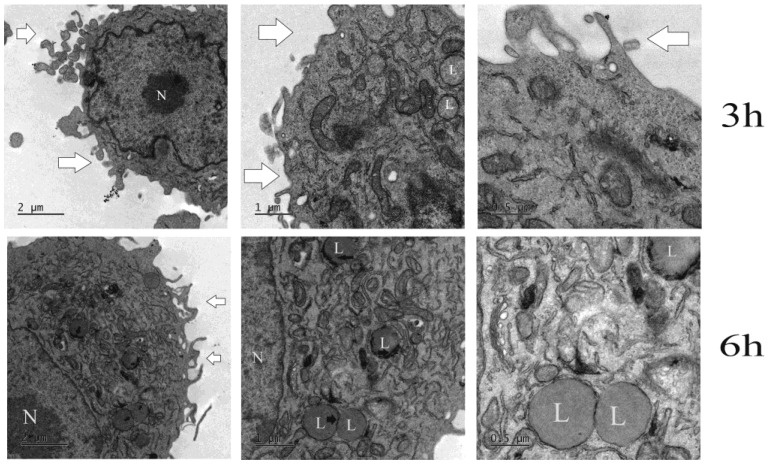
Transmission electron microscopy of MDA-MB-231 after 3 h and 6 h treatment with SLN-DTX. N indicates the nucleus, L the lysosomes, black arrows in the first image show the electrodense area on the lysosome where the presence of NLS-DTX can be observed, and the white arrows demonstrate the extensions of the cell membranes, which were still in the early stages of treatment.

Nanocarriers demonstrate increased cellular uptake upon binding to the cell membrane and enter the cytosol by endocytosis [23]. When SLN-DTX is internalized, it is directed to the lysosome, as seen in Figure 7, where SLN-DTX entered the cell in a different way than DTX. In the period of 3 h, it was possible to observe the lysosomes, and 6 h after the treatment there was the formation of several lysosomes with more electrodense particles, which may indicate the location of SLN-DTX because it contrasted with osmium tetroxide and bound to the lipid structure of the nanoparticle.

**Figure 8:**
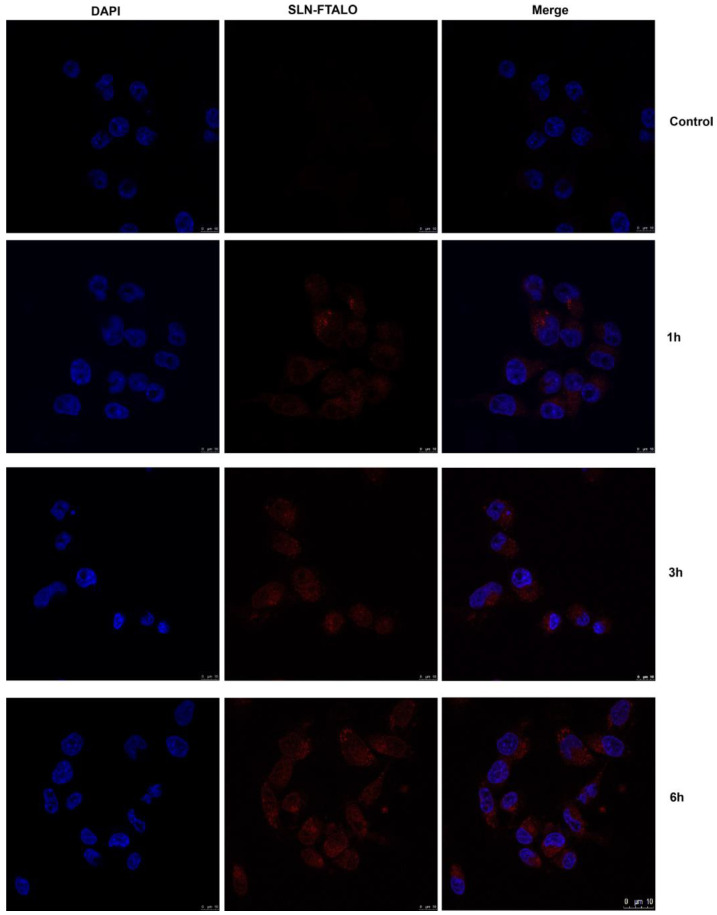
Confocal microscopy of MDA-MB-231 after 1 h, 3 h, and 6 h treatment with SLN-FTALO and control cells. DAPI blue stained the nucleus of the cells, while red shows the SLN-FTALO accumulated in the lysosomes region. Scale bar 10 μm.

The location and time of internalization were also corroborated by the confocal microscopy with SLN-FTALO, as seen in Figure 8.

Small, hydrophobic molecules like DTX tend to enter the cell by simple diffusion, due to the solubility of the lipid bilayer. The differences in internalization may imply different interactions with the drug after its nanoparticle release. The drug’s time of action is different from when it is encapsulated, because even after 6 h of treatment the drug is found in the lysosome, indicating that the time for interaction with cellular structures is different from the free drug. It has been previously reported that SLNs can bind to the cell membrane and enter tumor cells which, in turn, increases the cellular uptake of these nanoparticles compared to the free solution. Furthermore, SLNs can accumulate and be retained within the tumor cells, while the free DTX solution diffuses and cannot accumulate in the cells [11,30,31,32].

### 2.6. Wound Healing

**Figure 9:**
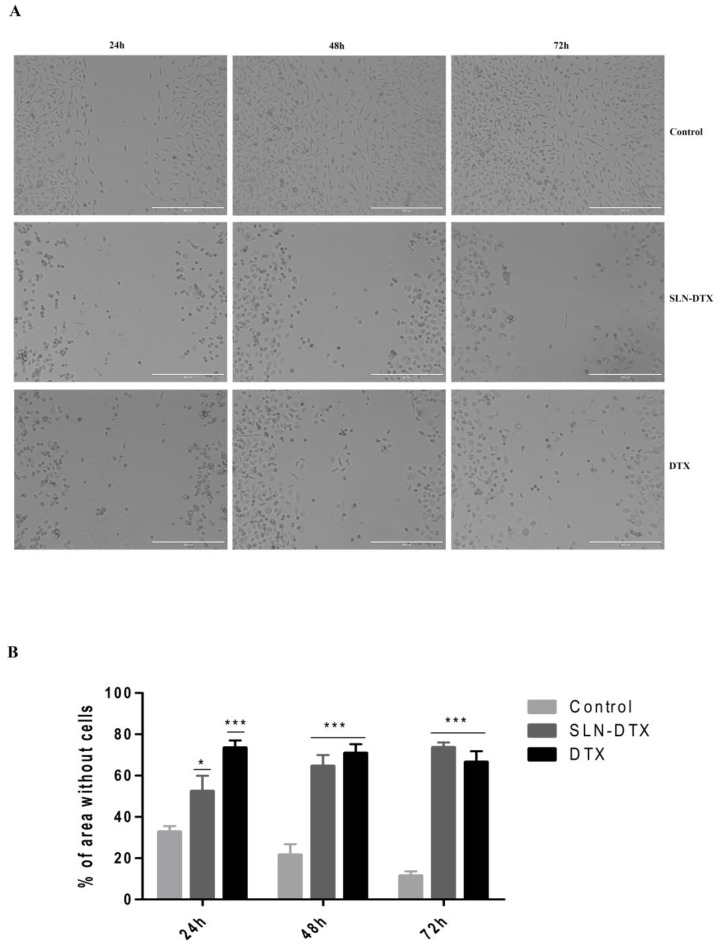
Analysis of wound healing of MDA-MB-231. (**A**) Light microscopy with control and the treatments with SLN-DTX and DTX, after 24 h, 48 h, and 72 h. Scale bar 400 μm. (**B**) Histogram with an area without cells for each one of the treatments with the different times. Area results in triplicate of each of the treatments. * *p* < 0.05 and *** *p* < 0.001 compared to untreated control.

Through the wound healing assay, it can be seen in Figure 9 that the treatments with SLN-DTX and DTX inhibited cell proliferation and mobility when compared to untreated cells. At the end of 72 h, the control group had already filled almost the entire area, while in both treatments the cells did not have the same feature even after 72 h. Additionally, when the empty area is quantified, it is clear that the treatments changed the ability of cells to move.

The wound healing assay was used to evaluate the proliferation and cell mobility capacity of the MDA-MB-231 cells using the comparison between the migration of cells with the different treatments (SLN-DTX and DTX) and with the control. Cells treated with SLN-DTX have the ability to inhibit proliferation and mobility in a similar way to those observed in treatment with DTX. Both proliferation and mobility are important factors for growth and enable cells to migrate to distant organs, so this inhibition can prevent metastatic tumor formation [33,34,35,36].

### 2.7. Cell Cycle Analyses

**Figure 10:**
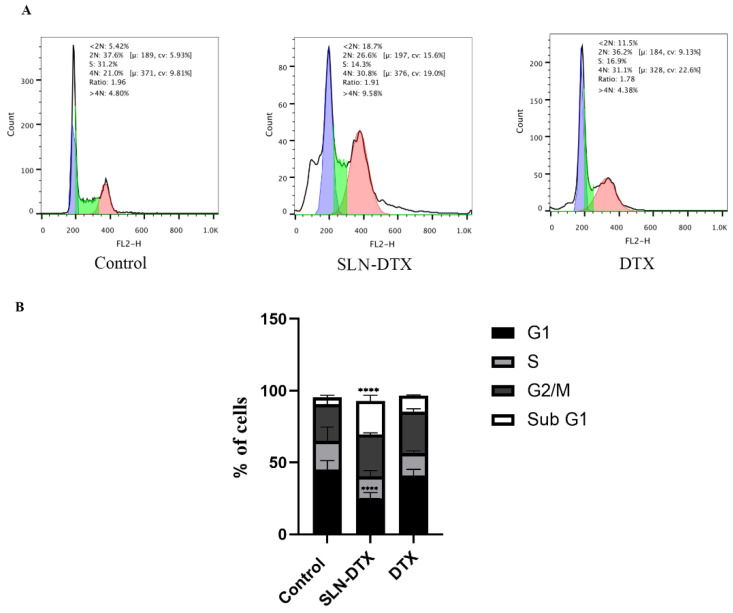
Cell cycle analysis of MDA-MB-231 after treatments. (**A**) Histograms of cell cycle phases, untreated cells, and cells treated with SLN-DTX and with DTX. Purple are cells in 2N, green are cells in S and pink are cells in 4N. (**B**) Quantification of the percentage of cells in each phase of the cell cycle. The results presented are from three independent experiments, with the mean percentage of cells in each cell cycle phase ± SEM. **** *p* < 0.0001 when compared to the control.

Antimitotic-induced cell death can occur both during mitotic arrest and after exiting the normal mitotic cycle, depending on the cell type and drug [26]. To assess whether treatments with NLS-DTX and DTX were interrupting cell cycle progress, MDA-MB-231 cells were labeled with PI and analyzed by flow cytometry, as shown in Figure 10.

In the group of cells treated with SLN-DTX, there was an increase in cells trapped in sub-G1 compared to the control, a characteristic seen for other nanoparticles such as nanoparticles with folate-targeted nanoparticle docetaxel [37], liposome-based co-delivery of siRNA and docetaxel [38], docetaxel-loaded mixed micelles, and polymersomes DTX [39], which could be linked to apoptosis of these cells. 

### 2.8. Cell Death Analyses

**Figure 11:**
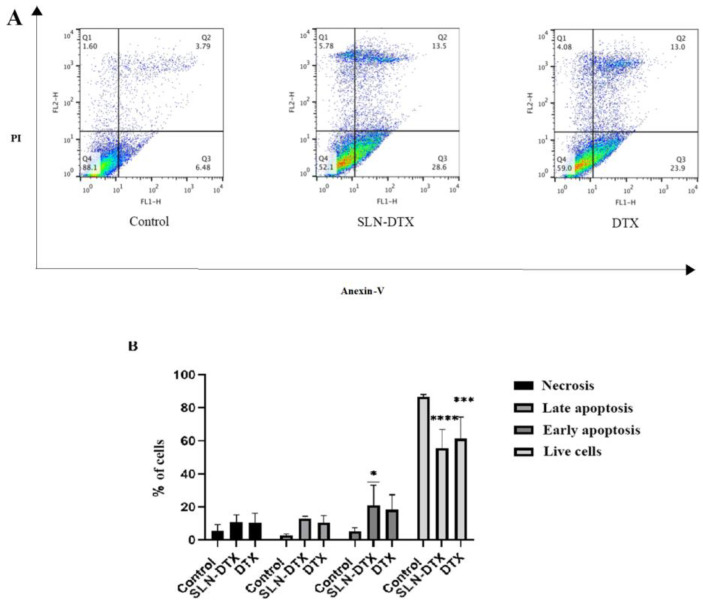
Analysis of cell death type. (**A**) Cell distribution according to the markings profile. Q1: necrotic cells, Q2: late apoptosis, Q3: early apoptosis, Q4: viable cells. The colors represent the population of cells, the warmer the color, the greater the number of cells in the region. (**B**) Histogram of cell death profiles by treatment with the mean + SEM of three independent experiments. * *p* < 0.05 *** *p* < 0.001 and **** *p* < 0.0001 compared to control.

Corroborating the cell cycle assay, the annexin/PI apoptosis assay showed a significant increase in apoptotic cells in the SLN-DTX treated group, as shown in Figure 11.

Other nanocarriers such as multi-walled carbon nanotubes conjugated with d-alpha-tocopherol polyethylene glycol 1000 succinate-loaded docetaxel [40] and docetaxel-loaded nanostructured lipid carriers [41] have a higher percentage of apoptotic cell death than free drugs, as they are more retained in the intracellular environment [40,41,42].

## 3. Materials and Methods

### 3.1. Materials

Dulbecco’s modified Eagle medium (DMEM) and fetal bovine serum (FBS) were purchased from Gibco. Leibovitz (L15); streptomycin; penicillin; DMSO; docetaxel; hematoxilin were purchased from Sigma-Aldrich (St. Louis, MO, USA). MTT (3-[4,5-dimethylthiazol-2-yl]-2,5 diphenyltetrazoliumbromide); propidium iodide; Anexina-V cj FITC; Alexa-488 were purchased from Invitrogen. Osmium tetroxide; Spurr resin were purchased from Electron Microscopy Sciences.

### 3.2. Methods

#### 3.2.1. Preparation of Solid Lipid Nanoparticles (SLNs)

The nanoparticles used were prepared according to the process described by Da Rocha et al. (2020) [21]. Solid lipid nanoparticles were prepared by the method of high energy. Compritol was selected as the solid lipid, and the surfactants selected were Span 80 and Pluronic F127; nanoparticles were divided into two different groups, solid lipid nanoparticles with docetaxel (SLN-DTX) and solid lipid nanoparticles without docetaxel (Blank-SLN).

#### 3.2.2. Cell Culture

MDA-MB-231 cell lines were acquired from the Cell Bank of Rio de Janeiro. These cells were cultured in Leibovitz medium (L15) at 37° in the absence of CO*X*_2_. The cell medium was supplemented with 10% fetal bovine serum (FBS) and with 1% antibiotic solution (100 units/mL of penicillin and 100 mg/mL of streptomycin).

#### 3.2.3. Cell Viability Assay

The cytotoxicity was determined by a colorimetric-based MTT assay. Cells were seeded in a 96-well plate at a density of 3 × 103 cells/well and incubated overnight. Then, each well was replaced with fresh medium containing 1 ng/mL, 10 ng/mL, 100 ng/mL, 1 µg/mL, 10 µg/mL, and 100 µg/mL of DTX, SLN-DTX, or culture medium (negative control) for 24, 48, or 72h. After the different incubation periods, the old medium was removed and MTT solution was added to each well, followed by incubation for 4 h at 37 °C to obtain formazan crystals. Finally, the cells and crystals were dissolved by adding dimethyl sulfoxide (DMSO) to each well followed by measuring the UV absorbance in the SpectraMax M5 (Molecular Devices, Sunnyvale, CA, USA) of these solutions at 595 nm to calculate the percentage of cell viability.

#### 3.2.4. Light Microscopy

To evaluate the morphological changes in MDA-MB-231 cells, 5 × 104 cells were seeded in 24-well plates, and the analyses were performed at 24 h, 48 h, and 72 h after treatments in concentrations of 1 µg/mL for each treatment (SLN-DTX and DTX). Analyses were performed by light microscopy on an Evos Microscope (Thermo Fisher Scientific, Waltham, MA, USA). To assess cell structures, 5 × 104 cells were seeded in round coverslips in 24-well plates; after 72 h of treatment with SLN-DTX and DTX at a concentration of 1 µg/mL, cells were fixed with 95% ethanol for 1 min and stained with hematoxylin for 1 min and eosin for 45 s. They were then washed with 95% ethanol for 1 min, followed by two washes with 100% ethanol for 1 min and two washes of xylene for 2 min, and were observed by light microscopy on an Axiophot Microscope (Zeiss, Oberkochen, Germany).

#### 3.2.5. SEM Microscopy

To visualize possible changes in cell shape and surface after treatments, cells were treated and analyzed under a scanning electron microscope (SEM). A total of 5 × 105 cells were seeded on 18x18mm coverslips placed at the bottom of the 6-well plate. After adhesion, the cells received the treatment for 72 h with SLN-DTX at concentrations of 1 µg/mL or did not. Then, the culture medium was discarded and the cells were washed twice with 1X PSB and then fixed with Karnovsky fixative solution (containing 2% glutaraldehyde, 2% paraformaldehyde, and 3% sucrose in sodium cacodylate buffer 0.1 M pH 7.2), overnight. The following day, cells were washed with 0.1 M sodium cacodylate buffer, pH 7.2. Coverslips were incubated in vapor of sodium tetroxide 2% osmium for 30 min and then washed with distilled water. Dehydration was carried out in increasing series with acetone (50–100%) and, finally, drying to a critical point using CPD 030 (BALZERS, Geneva, IL, USA) and SCD 500 metalization (LEICA, Wetzlar, Hesse, Germany), to be analyzed in a JSM 7001F scanning electron microscope (15 kV) (Jeol—Tokyo, Japan). 

#### 3.2.6. Immunostaining of Beta-Tubulin

To analyze the interference of treatment on the structure of the therapeutic target of docetaxel, beta-tubulin, the cells were labeled with an anti-*β*-tubulin antibody. 1 × 10^5^ MDA-MB-231 cells were seeded onto round coverslips in 24-well plates. After adhesion, the cells received or did not receive treatment for 72 h with DTX and SLN-DTX at a concentration of 1 µg/mL. After the treatment time, the cells were washed with PBS, fixed with 3.7% formaldehyde for 30 min, and permeabilized with 0.1% Triton X-100 for 20 min. The blocking solution (2.5% bovine serum albumin (BSA), 8% fetal bovine serum (SFB) in PBS) was added and left for 20 min, soon after, the cells were incubated with primary antibody of mouse anti-*β*-tubulin, overnight at 4 °C. The wells were washed with PBS and the secondary anti-mouse antibody Alexa-488 (5 g/mL) was added for 1 h at 37 °C protected from light. The wells were then washed with PBS and incubated for 5 min with DAPI (300 nM) to label cellular DNA. Then the wells were washed with PBS and the slides were mounted with ProLong Gold Antifade and analyzed using a TCS SP5 Scanning Confocal Laser Microscope 143 (Leica Microsystems, Wetzlar, Germany).

#### 3.2.7. TEM Microscopy

To observe the internalization of SLN-DTX, 1 × 10^6^ cells were seeded in 6-well plates and treated with SLN-DTX at a concentration of 1 µg/mL for 3 and 6 h. For the ultra-structural analysis caused by the treatment with the nanoparticle, 2 × 10^6^ cells were plated in 6-well plates and, after adhesion, the cells received treatment with SLN-DTX at a concentration of 1 µg/mL for 72 h, while the control did not receive any treatment. The cells were fixed for 1 h in Karnovsky’s fixative (containing 2% glutaraldehyde, 2% paraformaldehyde, and 3% sucrose in sodium cacodylate buffer 0.1 M pH 7.2). Subsequently, the cells were postfixed with 1% osmium tetroxide and 0.8% potassium ferricyanide in sodium cacodylate buffer 0.1 M pH 7.2 for 1 h. The material was washed with sodium cacodylate buffer and counterstained for 24 h with 0.5% uranyl acetate at 4 °C. Then, the cells were washed, dehydrated in increasing concentrations of acetone (30–100%), and embedded in Spurr resin. Ultrathin sections from 50 nm to 60 nm were obtained with diamond knives in an ultramicrotome (Leica Microsystems, Wien, Austria). The sections were mounted on copper screens and examined in a Jeol 1011 transmission electron microscope (Jeol, Peabody, MA, USA) at an acceleration voltage of 80 kV.

#### 3.2.8. Confocal Microscopy

To analyze the internalization of SLN in MDA-MB-231 cells, Blank-SLN was combined with aluminum chloride-phthalocyanine. This formulation was made exclusively for this experiment. A total of 1 × 10^5^ MDA-MB-231 cells were seeded onto round coverslips in 24-well plates. After adhesion, the cells were treated with SLN-FTALO (30 mM) for 1, 3, or 6 h, and the control did not receive any treatment. After the treatment time, the cells were washed with PBS and fixed with 3.7% formaldehyde for 30 min. The wells were then washed with PBS and incubated for 5 min with DAPI (300 nM) for labeling cellular DNA. Then the wells were washed with PBS and the slides were mounted with ProLong Gold Antifade and analyzed using a TCS SP5 scanning confocal laser microscope (Leica Microsystems, Germany).

#### 3.2.9. Wound Healing

To assess the ability of cells to move, the wound healing experiment was performed.MDA-MB-231 cells were plated in 24-well plates with 5 × 10^4^ cells per well. Then, the tip of a 200 µL tip was used to remove the cells in a uniform manner. Each well was washed 3 times with PBS and then three wells received treatments with 1 µg/mL of DTX and SLN-DTX or only the culture medium with 1% fetal bovine serum, as a control. After 24 h, 48 h, and 72 h, the images of each treatment were recorded on an Axiovert 100 Microscope (Zeiss, Germany). For image analysis, ImageJ Software was used to calculate the area without cells, and each time three images were used for each treatment.

#### 3.2.10. Cell Cycle

MDA-MB-231 cells were seeded with 1 × 10^5^ in 12-well plates. After their adhesion, the cells received treatment with SLN-DTX or docetaxel in the concentration of 1 µg/mL or only culture medium for 72 h. After the treatment period, the supernatant was collected, and cells were washed with PBS, trypsinized, and centrifuged. Then, the cell pellet from each group was washed with PBS and fixed with ice-cold 70% ethanol for 2 h at 4 °C. Cells were washed with PBS and resuspended in propidium iodide solution (10 µg/mL PI, 100 µg/mL DNAse free RNAse and 0.1% Triton X-100) diluted in PBS, for 10 min at 37 °C. After the incubation time, the samples were centrifuged and resuspended in PBS and 20,000 events from each sample were analyzed by FACSCalibur flow cytometry (Becton Dickinson, San Jose, CA, USA). Three independent experiments were performed.

#### 3.2.11. Cell Death

Annexin-V staining was performed to differentiate apoptosis from necrotic cell death induced by SLN-DTX or DTX. The cells were then harvested, washed with cold PBS, and centrifuged. The cell pellet was resuspended in annexin-binding buffer, and then annexin-V FITC conjugate and propidium iodide solution were sequentially added to the sample in order to detect apoptotic and necrotic cell populations, respectively. Finally, the cells were incubated at room temperature for 15 min and measurement was conducted by flow cytometer. A total of 20,000 events were collected per sample. Three independent experiments were performed.

#### 3.2.12. Statistical Analysis

Statistical evaluation of data was performed using a t-test and one or two-way ANOVA analyses of variance, followed by Tukey’s post-tests using GraphPad Prism software (version 5.0, created by Dr. Harvey Motulsky, San Diego, CA, USA). *p* < 0.05 was considered as the statistically significant difference. Quantitative results were expressed as mean ± standard deviation (SD).

## 4. Conclusions

Triple-negative breast cancer has a marked lack of treatments when compared to the other types of breast cancer, and the use of nanotechnology can improve therapies for this type of cancer. SLN-DTX represents a good candidate therapy for treating TNBC, because SLN-DTX maintained similar action to DTX in its main activities, proving that SLN is a good carrier to treat a very aggressive cell line, such as MDA-MB-231. SLN-DTX was also responsible for improving some activities when compared to conventional DTX treatment in the same concentrations and conditions, such as a greater induction of apoptosis and better sequestration of the cell cycle. The mitotic catastrophe observed after the treatment is an event that has not been well described in this type of cell, and further studies are necessary to understand its implication in the current treatment of breast cancer. The nanoparticle also has other advantages conferred by a nanocarrier to the drug, such as controlled and sustained release, better solubility, and stability, which could reduce the side effects seen in clinical practice and also reduce the number of applications given to the patient.

## Data Availability

The data presented in this study are available on request from the corresponding author.

## Sample Availability

Samples of the compounds are not available from the authors.

## References

[1] Nenclares P., Harrington K.J. (2020). The biology of cancer. Medicine.

[2] Hassanpour S.H., Dehghani M. (2017). Review of cancer from perspective of molecular. J. Cancer Res. Pract..

[3] Hussain A., Oves M., Alajmi M., Hussain I., Amir S., Ahmed J., Rehman M.T., El-Seedi H.R., Ali I. (2019). Biogenesis of ZnO nanoparticles using Pandanus odorifer leaf extract: Anticancer and antimicrobial activities. RSC Adv..

[4] da Silva J.L., Cardoso Nunes N.C., Izetti P., de Mesquita G.G., de Melo A.C. (2020). Triple negative breast cancer: A thorough review of biomarkers. Crit. Rev. Oncol. /Hematol..

[5] Yin L., Duan J.J., Bian X.W., Yu S.C. (2020). Triple-negative breast cancer molecular subtyping and treatment progress. Breast Cancer Res..

[6] Lehmann B.D., Bauer J.A., Chen X., Sanders M.E., Chakravarthy A.B., Shyr Y., Pietenpol J.A. (2011). Identification of human triple-negative breast cancer subtypes and preclinical models for selection of targeted therapies. J. Clin. Investig..

[7] Sharma P. (2016). Biology and Management of Patients with Triple-Negative Breast Cancer. Oncologist.

[8] Rodenak-Kladniew B., Islan G.A., de Bravo M.G., Durán N., Castro G.R. (2017). Design, characterization and in vitro evaluation of linalool-loaded solid lipid nanoparticles as potent tool in cancer therapy. Colloids Surf. B Biointerfaces.

[9] Sharma P., López-Tarruella S., García-Saenz J.A., Ward C., Connor C.S., Gómez H.L., Prat A., Moreno F., Jerez-Gilarranz Y., Barnadas A. (2017). Efficacy of Neoadjuvant Carboplatin plus Docetaxel in Triple-Negative Breast Cancer: Combined Analysis of Two Cohorts. Clin. Cancer Res..

[10] Martin M., Ramos-Medina R., Bernat R., García-Saenz J.A., del Monte-Millan M., Alvarez E., Cebollero M., Moreno F., Gonzalez-Haba E., Bueno O. (2021). Activity of docetaxel, carboplatin, and doxorubicin in patient-derived triple-negative breast cancer xenografts. Sci. Rep..

[11] Dawoud M. (2021). Chitosan coated solid lipid nanoparticles as promising carriers for docetaxel. J. Drug Deliv. Sci. Technol..

[12] Zhang E., Xing R., Liu S., Li P. (2019). Current advances in development of new docetaxel formulations. Expert Opin. Drug Deliv..

[13] Mukhtar E., Adhami V.M., Mukhtar H. (2014). Targeting Microtubules by Natural Agents for Cancer Therapy. Mol. Cancer Ther..

[14] Dumontet C., Jordan M.A. (2010). Microtubule-binding agents: A dynamic field of cancer therapeutics. Nat. Rev. Drug Discov..

[15] Chen R., Ni S., Chen W., Liu M., Feng J., Hu K. (2021). Improved Anti-Triple Negative Breast Cancer Effects of Docetaxel by RGD-Modified Lipid-Core Micelles. Int. J. Nanomed..

[16] Müller R.H., Mäder K., Gohla S. (2000). Solid lipid nanoparticles (SLN) for controlled drug delivery—A review of the state of the art. Eur. J. Pharm. Biopharm..

[17] Akanda M., Getti G., Nandi U., Mithu M.S., Douroumis D. (2021). Bioconjugated solid lipid nanoparticles (SLNs) for targeted prostate cancer therapy. Int. J. Pharm..

[18] García-Pinel B., Porras-Alcalá C., Ortega-Rodríguez A., Sarabia F., Prados J., Melguizo C., López-Romero J.M. (2019). Lipid-Based Nanoparticles: Application and Recent Advances in Cancer Treatment. Nanomaterials.

[19] Wang W., Chen T., Xu H., Ren B., Cheng X., Qi R., Liu H., Wang Y., Yan L., Chen S. (2018). Curcumin-Loaded Solid Lipid Nanoparticles Enhanced Anticancer Efficiency in Breast Cancer. Molecules.

[20] Amreddy N., Babu A., Muralidharan R., Panneerselvam J., Srivastava A., Ahmed R., Mehta M., Munshi A., Ramesh R. (2018). Recent advances in nanoparticle-based Cancer Drug and Gene Delivery. Adv. Cancer Res..

[21] da Rocha M.C.O., da Silva P.B., Radicchi M.A., Andrade B.Y.G., de Oliveira J.V., Venus T., Merker C., Estrela-Lopis I., Longo J.P.F., Báo S.N. (2020). Docetaxel-loaded solid lipid nanoparticles prevent tumor growth and lung metastasis of 4T1 murine mammary carcinoma cells. J. Nanobiotechnol..

[22] Kaushik L., Srivastava S., Panjeta A., Chaudhari D., Ghadi R., Kuche K., Malik R., Preet S., Jain S., Raza K. (2020). Exploration of docetaxel palmitate and its solid lipid nanoparticles as a novel option for alleviating the rising concern of multi-drug resistance. Int. J. Pharm..

[23] Qureshi O.S., Kim H.S., Zeb A., Choi J.-S., Kim H.-S., Kwon J.-E., Kim M.-S., Kang J.-H., Ryou C.-S., Park J.-S. (2017). Sustained release docetaxel-incorporated lipid nanoparticles with improved pharmacokinetics for oral and parenteral administration. J. Microencapsul..

[24] Mann J., Yang N., Montpetit R., Kirschenman R., Lemieux H., Goping I.S. (2020). BAD sensitizes breast cancer cells to docetaxel with increased mitotic arrest and necroptosis. Sci. Rep..

[25] Gadadhar S., Bodakuntla S., Natarajan K., Janke C. (2017). The tubulin code at a glance. J. Cell Sci..

[26] Shi J., Mitchison T.J. (2017). Cell death response to anti-mitotic drug treatment in cell culture, mouse tumor model and the clinic. Endocr.-Relat. Cancer.

[27] Tischer J., Gergely F. (2019). Anti-mitotic therapies in cancer. J. Cell Biol..

[28] Vitale I., Galluzzi L., Castedo M., Kroemer G. (2011). Mitotic catastrophe: A mechanism for avoiding genomic instability. Nat. Rev. Mol. Cell Biol..

[29] Janssen A., Kops G.J.P.L., Medema R.H. (2009). Elevating the frequency of chromosome missegregation as a strategy to kill tumor cells. Proc. Natl. Acad. Sci. USA.

[30] Zhang R., Qin X., Kong F., Chen P., Pan G. (2019). Improving cellular uptake of therapeutic entities through interaction with components of cell membrane. Drug Deliv..

[31] Zhang F., Chang M., Yu Y., Zhang Y., Liu G., Wei T., Zuo T., Guan Y., Lin G., Zhao Z. (2015). Preparation and evaluation of lipid emulsified docetaxel-loaded nanoparticles. J. Pharm. Pharmacol..

[32] Jadon R.S., Sharma M. (2019). Docetaxel-loaded lipid-polymer hybrid nanoparticles for breast cancer therapeutics. J. Drug Deliv. Sci. Technol..

[33] Tao J., Tan Z., Diao L., Ji Z., Zhu J., Chen W., Hu Y. (2018). Co-delivery of dihydroartemisinin and docetaxel in pH-sensitive nanoparticles for treating metastatic breast cancer via the NF-κB/MMP-2 signal pathway. RSC Adv..

[34] Li N., Guo W., Li Y., Zuo H., Zhang H., Wang Z., Zhao Y., Yang F., Ren G., Zhang S. (2020). Construction and anti-tumor activities of disulfide-linked docetaxeldihydroartemisinin nanoconjugates. Colloids Surf. B Biointerfaces.

[35] Li J., Zhang J., Wang Y., Liang X., Wusiman Z., Yin Y., Shen Q. (2017). Synergistic inhibition of migration and invasion of breast cancer cells by dual docetaxel/quercetin-loaded nanoparticles via Akt/MMP-9 pathway. Int. J. Pharm..

[36] Li G., Wang Y., Li L., Ren Y., Deng X., Liu J., Wang W., Luo M., Liu S., Chen J. (2020). Design, synthesis, and bioevaluation of pyrazolo[1,5-a]pyrimidine derivatives as tubulin polymerization inhibitors targeting the colchicine binding site with potent anticancer activities. Eur. J. Med. Chem..

[37] Wang E.C., Sinnott R., Werner M.E., Sethi M., Whitehurst A.W., Wang A.Z. (2014). Differential cell responses to nanoparticle docetaxel and small molecule docetaxel at a sub-therapeutic dose range. Nanomed. Nanotechnol. Biol. Med..

[38] Qu M.-H., Zeng R.-F., Fang S., Dai Q.-S., Li H.-P., Long J.-T. (2014). Liposome-based co-delivery of siRNA and docetaxel for the synergistic treatment of lung cancer. Int. J. Pharm..

[39] Khodaverdi E., Tayarani-Najaran Z., Minbashi E., Alibolandi M., Hosseini J., Sepahi S., Kamali H., Hadizadeh F. (2019). Docetaxel-Loaded Mixed Micelles and Polymersomes Composed of Poly (caprolactone)-Poly (ethylene glycol) (PEG-PCL) and Poly (lactic acid)-Poly (ethylene glycol) (PEG-PLA): Preparation and In-vitro Characterization. Iran. J. Pharm. Res. IJPR.

[40] Singh R.P., Sharma G., Singh S., Kumar M., Pandey B.L., Koch B., Muthu M.S. (2016). Vitamin E TPGS conjugated carbon nanotubes improved efficacy of docetaxel with safety for lung cancer treatment. Colloids Surf. B Biointerfaces.

[41] Liu D., Liu Z., Wang L., Zhang C., Zhang N. (2011). Nanostructured lipid carriers as novel carrier for parenteral delivery of docetaxel. Colloids Surf. B Biointerfaces.

[42] Maroufi N.F., Vahedian V., Mazrakhondi S.A.M., Kooti W., Khiavy H.A., Bazzaz R., Ramezani F., Pirouzpanah S.M., Ghorbani M., Akbarzadeh M. (2020). Sensitization of MDA-MBA-231 breast cancer cell to docetaxel bymyricetin loaded into biocompatible lipid nanoparticles via sub-G1 cell cycle arrest mechanism. Naunyn-Schmiedeberg’s Arch. Pharmacol..

